# Probiotic properties and antimicrobial evaluation of silymarin-enriched *Lactobacillus* bacteria isolated from traditional curd

**DOI:** 10.1038/s41598-023-37350-3

**Published:** 2023-07-05

**Authors:** Babak Haghshenas, Amir Kiani, Saeideh Mansoori, Ehsan Mohammadi-noori, Yousef Nami

**Affiliations:** 1 Regenerative Medicine Research Center (RMRC), Health Technology Institute, Kermanshah University of Medical Sciences, Kermanshah, Iran; 2 Students Research Committee, Kermanshah University of Medical Sciences, Kermanshah, Iran; 3 Department of Food Biotechnology, Branch for Northwest and West Region, Agricultural Biotechnology Research Institute of Iran, Agricultural Research, Education and Extension Organization (AREEO), Tabriz, Iran

**Keywords:** Microbiology, Applied microbiology

## Abstract

Nowadays, the increasing use of medicinal plants in the treatment and prevention of diseases has attracted the attention of researchers. The aim of this work was to investigate the probiotic properties and antibacterial and antifungal activity of silymarin-enriched *Lactobacillus* bacteria against several important pathogenic bacteria and also *Aspergillus flavus* as one of the harmful molds in the food and health industries. For this purpose, 52 g-positive and catalase-negative bacteria were isolated from 60 traditional curd samples from Ilam province. Five of the 52 bacterial strains had more than 90% viability in high bile salt and acidic conditions and were selected for further investigation. The five strains with positive results showed good hydrophobicity (≥ 50.30%), auto-aggregation (≥ 53.70%), coaggregation (≥ 28.20%), and high cholesterol removal ability (from 09.20 to 67.20%) and therefore can be considered potential probiotics. The tested strains displayed acceptable antibacterial and antifungal activity against all 12 pathogenic bacteria and *A. flavus*. Also, the results of the simultaneous antifungal activity of probiotic strains and silymarin showed that the combination of silymarin and probiotics has a significantly better (*P* < 0.05) antifungal effect than the control group or the probiotic groups alone. Interestingly, in addition to the *Limosilactobacillus fermentum* C3 strain, the *Limosilactobacillus fermentum* C18 and *Lactiplantibacillus pentosus* C20 strains also had significant inhibitory effects against *A. flavus* when used with silymarin extract in methanol. Meanwhile, silymarin extract in DMSO and PEG increased the antagonistic activity of all five potential probiotic strains.

## Introduction

Molds that belong to the fungus group are microorganisms that generate numerous benefits and drawbacks in the food and beverage industries. *Aspergillus* is a group of hundreds of distinct forms of fungi that inhabit a variety of climates across the globe. Pier Antonio Micheli, an Italian cleric and biologist, was the first individual to enumerate *Aspergillus*. While observing the fungus under a microscope, Micheli named the genus after recalling the shape of an aspergillum (sacred water sprinkler) from the Latin word sparger (sprinkle). Aspergillum is an *Aspergillus*-specific asexual spore-forming structure. Approximately one-third of all species are sexually mature. Some species of *Aspergillus* have been linked to fungal infections, whereas others have commercial value^1^.

Researchers are currently interested in the expanding use of medicinal plants in the treatment and prevention of disease. The milk thistle plant, known scientifically as Marianum silybum, is also known as Maritigal, Khar Alis, and Akkub in Persian and Arabic^2^. It is biennial, glabrous, dull green, prickly, and has erect stems. The comparatively dense limbs of this basic or slightly branched plant lead to a verdant mound with horizontal channels. This plant's seed extract contains a variety of chemicals, including silybin A and B, silydianin, silychristin, apogenins, di-hydrosilybin, deoxysilychristin, and deoxysilydianin. The dry seed extract of the plant contains 1–4% silymarin, which is rich in flavonoids like silybin A and B, slydianin, silychristin, and dihydrosilybin^3^. This plant extract also contains cylandrin, silybin, silyhermine, myristic, palmitic, and stearic acids, which may have hepatoprotective effects^4^. In addition, the dry seed of the plant contains 15–20% oil, which has no medicinal properties^3^. After ingestion, the body absorbs 20–50% of the plant's seed extract. But the phosphatidyl-choline complex has a higher absorption^5^.

Most probiotic bacteria belong to the category of lactic acid bacteria (LAB). They are an order of gram-positive, low-GC, acid-tolerant, nonsporulating, nonrespiring, rod-shaped (bacilli) or spherical (cocci) bacteria with similar metabolic and physiological characteristics. Significant LAB microorganisms produce lactic acid as a consequence of their metabolic processes. The role of LAB in agriculture, nutrition, and clinical practice is multifaceted. Because they naturally produce bacteriocins that aid in food biopreservation, LAB serve as an antagonistic, inhibitory, and antibacterial defense mechanism against pathogens and spoilage microorganisms^6,7^. Probiotics are beneficial microorganisms that, when consumed in sufficient quantities, are beneficial to the recipient^8,9^. Similar to LAB, probiotics enhance and maintain the immune system of humans. For instance, it has been suggested that prescribing probiotics and LAB can prevent a variety of health-related disorders. Therefore, probiotics and lactic acid-fermented foods are nutritionally advantageous for consumers and function as immune system boosters against diseases and infections^10,11^.

One of the important concerns in the food industry is reducing the burden and economic losses caused by mold contamination. In addition to irreparable economic damage, mold in food causes the production of aflatoxins, which cause many problems for people's health in society. Aflatoxin B1 is one of the most potent liver carcinogens and is produced by a large number of *Aspergillus* species, especially *Aspergillus flavus*. Lactic acid bacteria may create organic acids, hydrogen peroxide, and bacteriocins, which are powerful against a wide range of infections. As a result, one of the most important methods of food preservation, the use of microorganisms with antagonistic and harmless effects, such as probiotics, to reduce the growth of *Aspergillus* mold and thus reduce the production of aflatoxin, is widely used to prevent and treat liver disease caused by aflatoxin. It has no harmful effects and is additionally used as a dietary supplement to enhance liver function. Recent reports suggest that silymarin has antibacterial properties against several harmful pathogens. Silymarin has an antifungal effect by increasing permeability and causing oxidative stress in a targeted manner via the membrane. Therefore, the goal of this work was to examine the independent and concurrent effects of silymarin and a putative probiotic, *Lactobacillus* extract, on *A. flavus*.

## Materials and methods

### Sample collection

A total of 60 homemade curd samples were gathered from different parts of the Ilam Province of Iran. The samples were brought in ice boxes to the lab and kept there at 4 °C. Initial homogenization of the curd was done using a vortex in order to better separate the bacteria from the solid curd particles. To enhance and strengthen the initial bacterial community, 5 g of each sample was added to 10 mL of de Man Rogosa Sharpe (MRS) broth and grown for 48 h at 37 °C under anaerobic conditions. The clear colonies were then chosen for analysis after 100 µL of each sample was grown on MRS agar medium and incubated at 37 °C for 24 h. Gram staining, catalase activity, and cell morphology were performed before selecting Gram-positive and catalase-negative cells for further investigation^6^.

### Probiotic characterization

#### Low pH and high bile salt tolerance

An acid and bile resistance experiment was performed to select the most promising probiotic strains for further study. Acid tolerance was assessed based on the procedure of Kiani et al. with some modifications^12^. In brief, 10 mL of MRS broth supplemented with 100 µL of each strain (1.9–3.8 10^9^ CFU mL^−1^) was incubated for 24 h at 37 °C. After 5 min of centrifugation at 4500*g*, the cell aggregates were resuspended in PBS (phosphate-buffered saline) solution (pH 2.5) and maintained at 37 °C with gentle stirring for 3 h. The optical density (OD) data at 600 nm were used to calculate the resistance to low pH. The formula for calculating acid resistance is as follows:

$$\mathrm{Survival\,rate }\left(\mathrm{SR\%}\right)=\frac{\mathrm{OD }\left(\mathrm{after\,treatment}\right)}{\mathrm{OD }(\mathrm{before\,treatment})}\times 100$$

The approach presented by Kiani et al. was used to calculate resistance to high bile salt concentrations. In brief, 100 µL of strains were inoculated with 10 mL of MRS broth culture medium with 0.3% bile oxgall for 4 h at 37 °C. The OD at 620 nm was detected using a spectrophotometer (Eppendorf, Germany). The aforementioned formula was used to calculate the percentage of bile salt resistance^12^.

#### Survival under a simulated human GI tract condition

Strains with a high tolerance to highly acidic conditions and high bile salt content were chosen for further investigation. The formula presented by Kouhi and colleagues in 2021 was used to assess the survival rates. One percent of the selected strains were inoculated in MRS medium and incubated at 37 °C for 24 h. One mL of cultures was centrifuged at 4500×*g* for 5 min at 4 °C to obtain cell pellets (10^9^ CFU mL^−1^). The cells were reconstituted in 9 mL of PBS (pH 3) consisting of 3 mg mL^−1^ of pepsin enzyme from Sigma-Aldrich (St. Louis, Missouri, USA) and incubated for 3 h. The suspensions were centrifuged once more and then suspended in the physiological solution after being cleaned with sterile PBS. After performing proper serial dilutions, the viable cell counts were determined on the MRS agar plates and incubated at 37 °C in anaerobic conditions for 24 h. The selected isolates were introduced to digestive fluid containing 0.3% bile salts (Sigma-Aldrich) and 1 mg mL^−1^ pancreatin (Sigma-Aldrich), which was then adjusted to pH 8 and kept for 4 h at 37 °C. The strains' survival was assessed by counting the cells on MRS agar plates^6^.

#### Cell surface properties

##### Hydrophobicity

The adherence ability of strains to xylene and toluene was assessed based on the procedure outlined by Mishra and Prasad. Selected strains' overnight cultures were harvested by centrifuging at 4500×*g* for 5 min. After dispersing the pellets (10^9^ CFU mL^−1^) in 3 mL of PBS, the initial absorbance at 600 nm (A_0_) was measured. They were then allowed to come into contact with 1 mL of xylene and toluene (Merck, Germany) after whirling in a vortex for two minutes. The phases were separated by decantation for 1 h at 37 °C, and the aqueous phase's absorbance was determined (A_1_). The following equation was used to obtain the cell surface hydrophobicity rates^13^.

$${\text{Hydrophobicity }}\left( \% \right) = \left[ {{1} - \left( {{\text{A}}_{{1}} /{\text{A}}_{0} } \right) \times {1}00} \right]$$

##### Autoaggregation

The strains' aggregation activity was tested using the method presented by Angmo et al.^14^. In brief, strains (10^9^ CFU mL^−1^) were harvested after 24 h of incubation by centrifugation at 4500×*g* for 5 min, washed in sterile PBS, and suspended in the same buffer. Without agitation, the mixture was kept at 37 °C for 4 h^14^. The autoaggregation percentage was calculated using the formula below:

$$\mathrm{Auto}-\mathrm{aggregation\,}(\mathrm{\%}) =1- \frac{\mathrm{A}\left(\mathrm{t}\right)}{\mathrm{A}\left(0\right)} \times 100$$

where A_0_ denotes absorbance at the start and A_t_ denotes absorbance at time t.

##### Coaggregation

After 4 h of incubation at 37 °C, the coaggregation activities of strains against *Staphylococcus aureus* and *Bacillus cereus* were evaluated according to the procedure reported by Zuo et al.^15^. Briefly, equal volumes of suspension from different strains and two pathogens were incubated together for 4 h at 37 °C without agitation. The coaggregation percentage was determined using the equation below:

$${\text{Co}} - {\text{aggregation }}\left( \% \right) \, = \, \left[ {\left( {{\text{A}}_{p} + {\text{A}}_{i} } \right)/2 - \left( {{\text{A}}_{mix} } \right)/\left( {{\text{A}}_{p} + {\text{A}}_{i} } \right)/2} \right] \times 100,$$

where A_p_, A_i_, and A_mix_ denote the absorbance of pathogenic bacteria, selected strains, and their mixture after 4 h incubation.

#### Cholesterol assimilation

The cholesterol assimilation ability of strains was assessed based on the o-phthalaldehyde technique reported by Rudel and Morris with some modifications^16^. Each strain was put in a freshly prepared MRS broth containing 0.3 oxgall (Merck, Germany) as a bile salt and 150 mg mL^−1^ water-soluble cholesterol as a cholesterol source. The combination was then sterilized through 0.2 µL filters before being incubated anaerobically for 20 h at 37 °C. Following the incubation period, the cells were centrifuged (6000×*g* for 15 min) to remove them. One mL of cell-free supernatant was then mixed with one mL of KOH (33% w/v) and two mL of ethanol (96%), vortexed for two minutes, and heated at 60 °C for 15 min. After cooling to ambient temperature, the mixtures were treated with deionized water (2 mL) and hexane (3 mL) and shaken for 1 min. In a fresh glass tube, the hexane layer was vaporized for one mL in an 80 °C water immersion. The residue was quickly dissolved in 0.5 mL of concentrated sulfuric acid and 2 mL of o-phthalaldehyde (Merck, Germany) reagent before being vortexed for 1 min. After 30 min of room temperature incubation, the samples were tested for absorbance at 550 nm.

#### Antimicrobial activity of strains

The inhibitory metabolites produced by the bacteria tested were inferred and detected using a well-diffusion technique^10^. The chosen strains were grown in overnight cultures on MRS agar for 24 h at 37 °C. The study's indicator microorganisms, which were obtained from the Persian Type Culture Collection (PTCC), were *Streptococcus sanguinis* PTCC 1449, *Streptococcus salivarius* PTCC 1448, *Streptococcus sobrinus* PTCC 1601, *Yersinia enterocolitica* ATCC 23715, *Pseudomonas aeruginosa* PTCC 1181, *Staphylococcus aureus* ATCC 25923, *Streptococcus mutans* PTCC 1683, *Bacillus subtilis* ATCC 19652, *Klebsiella pneumoniae* PTCC 1053, *Listeria monocytogenes* ATCC 13932, *Shigella flexneri* PTCC 1234, and *Escherichia coli* PTCC 1276. Half a McFarland of indicator bacteria (1.5 10^8^ CFU mL^−1^) was poured into various appropriate media, and wells were cut into plates. The plates were then incubated overnight at 37 °C with each well filled with 50 µL filtered supernatant, and a digital caliper was used to estimate the inhibitory zone around the wells.

#### Antimicrobial activity of silymarin

The well-diffusion assay was used to assess the inhibitory effect of silymarin against indicator bacteria listed in section "Antifungal activity of selected strains". For this purpose, silymarin was diluted in DMSO and methanol separately. Different dilutions of silymarin were put in the wells created in MRS agar medium and allowed bacteria to grow for 24 h at 37 °C. The inhibition zone around the wells was then determined.

#### Antifungal activity of selected strains

The modified overlay technique was applied to assay the inhibitory effects of selected strains against *A. flavus*. MRS agar plates were prepared, and strains were streaked on the plates. After overnight incubation, soft potato dextrose agar (PDA, 0.7% agar) containing fungal spores of known inoculation size (2 × 10^5^ spores mL^−1^) was poured onto the MRS agar plates. After that, the plates underwent aerobic incubation for seven days at 30 °C. They were checked for distinct areas of inhibition around the bacterial streaks, which were referred to as positive effect zones ^17^.

#### Simultaneous antifungal activity of strains and silymarin

For this purpose, the most effective dilution of silymarin was selected and assessed with the extract of selected strains. First, MRS agar medium was prepared, and selected strains were cultured onto the plates and allowed to grow for 24 h^17^. Then, a soft PDA medium was prepared, and before the medium was frozen and poured into the plates, the most effective dilution of filtered silymarin was added to it. Soft PDA medium was put onto the MRS agar and incubated at 30 °C for seven days. Finally, the zone of inhibition around the streaked strains was calculated.

### Safety aspects

#### Hemolysis activity

Growing fresh overnight cultures on blood agar (Oxoid) consisting of 7% (v/v) sheep blood and incubating at 37 °C for 48 h determined the strains' hemolytic properties. Finally, hemolytic activity was divided into three groups based on the appearance of zones of inhibition around colonies: a greenish zone indicating α-hemolysis, a clear zone indicating β-hemolysis, and no zone of inhibition indicating γ-hemolysis^6^.

#### Antibiotic susceptibility

A disk-diffusion procedure was performed to ascertain the antibiotic susceptibility characteristics of the strains. Antibiotics evaluated included cefixime (15 mg), azithromycin (30 mg), amoxicillin (20 mg), doxycycline (15 mg), trimethoprim-sulfamethoxazole (30 mg), ciprofloxacin (20 mg), cephalexin (30 mg), amoxicillin-clavulanic acid (30 mg), and vancomycin (30 mg). After being kept at 37 °C for one night, the diameter of inhibition (in millimeters) around each disk was measured^18^.

### Molecular identification and DNA sequencing

Amplification of the 16S rRNA gene (1544 bp) of the strains was done using pair primers Hal6F: 5′-AGAGTTTGATCMTGGCTCAG-3′ and Hal6R: 5′-TACCTTGTTAGGACTTCACC-3′. The PCR protocol was done based on the procedure presented by Nami et al.^19^. On a 1% agarose gel, the PCR products were electrophoresed and stained with ethidium bromide. The Macrogene DNA Sequencing Service sequenced the PCR products (Korea). Following analysis, the sequences of the produced PCR products were matched with Clustal W and compared to isolates from the GenBank libraries.

### Statistical analysis

SPSS was used to perform statistical analysis on the data (Ver. 19.0 SPSS, Chicago, IL, USA). A one-way ANOVA with a significance level of *P* < 0.05 was employed to compare variations in treatment means. All tests were carried out in three replicates, and the data were presented as the mean plus standard deviations.

## Results and discussion

### Isolation and phenotypic identification

A total of 52 different round and cream-colored bacterial colonies containing rod- and cocci-shaped bacteria were grown on the culture medium and identified as LAB isolates using gram-positive and catalase-negative assays. These colonies were isolated from 60 traditional curd samples from Ilam province, Iran. The isolated strains were purified and stored in MRS broth containing 50% glycerol at 80 °C.

### Probiotic characterization

#### Low pH and high bile salt tolerance

The survival percentages of the strains after 3 h at pH 2.5 and 4 h at 0.3% bile were 9.44–99.21% and 16.08–100.00%, respectively. Based on the results presented in Table 1, the survival rates of 24 out of 52 strains were less than 50%, and 23 strains showed survival rates between 50 and 90% after 3 h of incubation at pH 2.5. In addition, 18 strains showed survival rates of less than 50%, and 29 strains had survival rates between 50 and 90% after 4 h of incubation at 0.3% bile salts. Only five strains (C3, C18, C20, C39, and C58) demonstrated greater than 90% low pH and high bile tolerance with survival rates of 96.60, 98.22, 97.52, 99.21, and 97.10%, respectively, at pH 2.5. All five strains showed 100% survival rates at 0.3 bile salts. As a consequence, these five strains were chosen for further study.

**Table 1:** Survival rates (%) of isolated LAB strains after 3 h incubation at pH 2.5 and 0.3% bile.

Survival rates at					
Isolated strain	pH 2.5	0.3% bile	Isolated strain	pH 2.5	0.3% bile
C1	37.24 ± 1.50^l^	42.63 ± 2.16^m^	C30	34.14 ± 0.83^m^	43.35 ± 1.01^m^
C2	33.82 ± 0.71^m^	42.18 ± 0.66^m^	C33	36.93 ± 0.44^l^	43.98 ± 0.34^m^
C3	96.60 ± 0.88^b^	100.00 ± 1.23^a^	C34	12.97 ± 0.21^o^	23.53 ± 0.59^n^
C4	12.33 ± 0.47^o^	23.24 ± 0.77^n^	C38	48.76 ± 0.44^j^	53.29 ± 0.72^k^
C5	18.94 ± 0.46^n^	23.01 ± 0.65^n^	C39	99.21 ± 1.93^a^	100.00 ± 1.91^a^
C6	52.76 ± 0.32^i^	63.17 ± 0.23^i^	C40	09.44 ± 0.14^p^	16.08 ± 0.77^o^
C7	48.56 ± 0.81^j^	54.09 ± 0.66^k^	C41a	63.08 ± 0.34^g^	70.12 ± 0.48^h^
C9	83.76 ± 0.94^c^	88.58 ± 0.22^ef^	C41b	77.83 ± 0.49^e^	86.93 ± 0.97^f^
C10	49.59 ± 0.87^j^	59.15 ± 1.18^j^	C42	09.85 ± 0.55^p^	16.22 ± 0.56^o^
C12	40.18 ± 1.34^k^	49.18 ± 1.23^l^	C43	48.76 ± 0.24^j^	53.79 ± 1.26^k^
C13	73.22 ± 0.67^f^	83.77 ± 0.57^g^	C44a	83.97 ± 0.88^c^	89.08 ± 0.44^ef^
C14	81.26 ± 0.78^d^	89.13 ± 0.93^e^	C44b	51.94 ± 0.68^i^	63.26 ± 1.05^i^
C15	63.50 ± 1.04^g^	70.60 ± 1.04^h^	C45	81.56 ± 0.72^d^	89.99 ± 0.38^e^
C16	37.46 ± 0.47^l^	42.55 ± 0.64^m^	C47	12.69 ± 0.47^o^	23.12 ± 0.32^n^
C17	78.38 ± 0.95^e^	86.70 ± 1.45^f^	C48	52.60 ± 0.91^i^	62.91 ± 0.41^i^
C18	98.22 ± 1.97^b^	100.00 ± 2.20^a^	C50	48.90 ± 0.50^j^	54.34 ± 1.03^k^
C20	97.52 ± 1.44^b^	100.00 ± 1.20^a^	C53	84.04 ± 0.42^c^	88.69 ± 0.56^ef^
C21	72.78 ± 1.12^f^	84.12 ± 1.97^g^	C54	72.66 ± 0.49^f^	84.36 ± 0.29^g^
C22	40.63 ± 0.94^k^	49.71 ± 0.84^l^	C55	77.94 ± 0.58^e^	86.97 ± 1.08^f^
C23	56.48 ± 0.39^h^	68.39 ± 1.03^h^	C56	63.26 ± 0.52^g^	70.49 ± 0.57^h^
C24a	34.57 ± 0.46^m^	43.57 ± 0.77^m^	C57a	73.70 ± 1.12^f^	83.27 ± 1.22^g^
C24b	49.18 ± 0.59^j^	59.28 ± 1.22^j^	C57b	40.39 ± 0.52^k^	48.69 ± 0.94^l^
C25	40.37 ± 1.13^k^	49.96 ± 1.12^l^	C58	97.10 ± 1.05^b^	100.00 ± 1.77^a^
C26	09.57 ± 0.21^p^	16.63 ± 0.28^o^	C59a	53.09 ± 0.67^i^	63.29 ± 1.14^i^
C28	13.07 ± 0.73^o^	23.72 ± 0.56^n^	C59b	78.12 ± 0.26^e^	87.44 ± 0.86^f^
C29	72.91 ± 1.44^f^	84.12 ± 1.97^g^	C60	56.72 ± 0.55^h^	68.87 ± 0.37^h^

^a–p^: Means with the same letter are not significantly different for each isolated strains (*P* < 0.05) in acid resistance assay.

^a–o^: Means with the same letter are not significantly different for each isolated strains (*P* < 0.05) in bile resistance assay.

Table 2 shows the results of the viability of selected strains under gastric and intestinal conditions. The results showed that the selected bacteria indicated high longevity in the early hours of incubation. Under gastric conditions, a slight decrease in the amount of logarithmic CFU mL^−1^ was observed after 1 h, but a further decrease was observed between 1 and 2 h. Table 2 shows that at the start of the in vitro digestion, the average counts ranged from 9.118 ± 0.12 to 9.717 ± 0.14 Log CFU mL^−1^. By the end of the gastric condition, the strains C3, C18, C20, C39, and C58 showed viable numbers of 6.298 ± 0.10, 6.916 ± 0.12, 5.420 ± 0.15, 6.161 ± 0.16, and 5.692 ± 0.14 Log CFU mL^−1^, respectively. pH is an important factor influencing probiotic growth and viability during transit through the stomach. When isolates from yogurt-like products^20^ and camel milk^21^ were subjected to low pH, a similar reduction trend was observed. Under intestinal conditions, good viability was observed for all five strains in the first hour, but a decrease in log CFU mL^−1^ was observed between 2 and 3 h. By the end of the intestinal condition, the strains C3, C18, C20, C39, and C58 had viable numbers of 7.192 ± 0.10, 7.487 ± 0.15, 6.521 ± 0.11, 6.455 ± 0.16, and 4.871 ± 0.10 Log CFU mL^−1^, respectively. These findings agree with other in vitro studies^18,22^. A strain of bacteria must have several important properties to be considered a probiotic. It should, for example, retain viability and activity during manufacturing, product storage, and transport through the gastrointestinal tract^23,24^. In general, the better the probiotic, the smaller the reduction in the number of viable bacteria after in vitro digestion^6,25^. The acid tolerance of all strains varied in the current study as a result of strain and/or species differences. The mechanism of resistance to GIT conditions varies depending on strain and species.

**Table 2:** Re-screening results and survival rates of the selected LAB strains after 2 h incubation in gastric condition (5% (w/v) pepsin at pH 2.5) and after 3 h incubation in intestinal condition (0.1% pancreatin and 0.3% bile at pH 6.0).

Isolated strain	Final counts (log CFU mL^−1^) after incubation at				Final counts (log CFU mL^−1^) after incubation at				
	0 h	1 h	2 h	SR (%) after 2 h	0 h	1 h	2 h	3 h	SR (%) after 3 h
C3	9.218 ± 0.15	8.934 ± 0.16	6.298 ± 0.10	68.32	9.719 ± 0.12	9.524 ± 0.14	7.418 ± 0.12	7.192 ± 0.10	74.00
C18	9.474 ± 0.13	9.123 ± 0.14	6.916 ± 0.12	73.00	9.573 ± 0.11	9.395 ± 0.17	7.653 ± 0.11	7.487 ± 0.15	78.21
C20	9.118 ± 0.12	8.664 ± 0.13	5.420 ± 0.15	59.44	9.847 ± 0.15	9.456 ± 0.12	6.917 ± 0.13	6.521 ± 0.11	66.22
C39	9.627 ± 0.10	9.207 ± 0.10	6.161 ± 0.16	63.40	9.214 ± 0.12	8.883 ± 0.10	6.848 ± 0.15	6.455 ± 0.16	70.06
C58	9.717 ± 0.14	8.624 ± 0.11	5.692 ± 0.14	58.58	9.734 ± 0.11	9.027 ± 0.16	5.539 ± 0.18	4.871 ± 0.10	50.04

SR: Survival rate.

#### Cell surface properties

Table 3 displays the outcomes of the hydrophobicity test. The findings revealed that the strains tested had cell surface hydrophobicity values greater than 50.30 ± 1.03 and 52.70 ± 1.79% with toluene and xylene, respectively. In addition, it was shown that strains C3 and C18 had the highest hydrophobic properties, with rates of 65.30 ± 2.08 and 62.10 ± 1.33% with xylene and toluene, respectively. These results confirm the results of several previous works reported by Mazlumi et al.^26^, Kiani et al.^23^ and Nami et al.^27^.

**Table 3:** Cholesterol uptake, Cell surface hydrophobicity, Adherence ability, Auto-aggregation (%), Co-aggregation (%), and Hemolytic activity of isolates tested. Values shown are means ± standard deviations (n = 3).

Isolates	Cholesterol uptake (%)	Hydrophobicity (%)		Auto-aggregation (%)	Co-aggregation (%)		Hemolytic activity
		Toluene	Xylene		*S. aureus*	*B. cereus*	
C3	67.20 ± 1.21^a^	62.10 ± 1.33^ab^	67.10 ± 1.10^a^	69.30 ± 1.10^a^	58.70 ± 1.10^a^	57.30 ± 1.85^a^	γ
C18	65.10 ± 1.10^a^	63.60 ± 1.50^a^	65.30 ± 2.08^a^	73.40 ± 1.33^a^	56.50 ± 1.33^a^	54.70 ± 0.98^a^	γ
C20	32.10 ± 1.04^b^	58.20 ± 1.79^bc^	59.10 ± 0.81^b^	64.20 ± 1.50^b^	34.30 ± 1.56^b^	33.80 ± 1.39^b^	γ
C39	13.40 ± 0.52^c^	50.30 ± 1.03^d^	52.70 ± 1.79^d^	55.90 ± 1.62^d^	32.10 ± 1.39^bc^	31.10 ± 1.50^b^	γ
C58	09.20 ± 0.64^d^	54.60 ± 1.27^cd^	55.90 ± 1.56^cd^	53.70 ± 1.10^d^	28.20 ± 0.92^c^	29.20 ± 1.21^b^	γ

*Values followed by the same letters are not significantly different (*P* < 0.05). Statistical analysis of each formulation was done separately.

The results obtained from the autoaggregation test are shown in Table 3. Autoaggregation was different among the strains obtained, and the highest accumulation was observed in the C18 and C3 strains with rates of 73.40 ± 1.33 and 69.30 ± 1.10%, respectively. Additionally, Table 3 displays the outcomes of the coaggregation of the separated strains. The results obtained showed that the highest rate of coaggregation in the presence of *S. aureus* and *B. cereus* bacteria was observed in strain C3, with rates of 58.70 ± 1.10 and 57.30 ± 1.85%, respectively. A similar aggregation ability trend was reported previously when strains were isolated from fermented foods and beverages from Ladakh^14^ and Motal cheese^6^.

For bacteria to adhere to the gut mucosa and colonize the GIT, cell surface characteristics such as cell surface hydrophobicity and autoaggregation are crucial. Coaggregation is an effective host defensive mechanism against the invasion of pathogenic microbes in the gastrointestinal system, and autoaggregation of probiotic bacteria is a necessary precondition for biofilm development^18^. Since they have these qualities, probiotics are useful ingredients in functional foods^6^. In the current study, strains C3 (62.10 ± 1.33 and 67.10 ± 1.10%), C18 (63.60 ± 1.50 and 65.30 ± 2.08%), C20 (58.20 ± 1.79 and 59.10 ± 0.81%), C39 (50.30 ± 1.03 and 52.70 ± 1.79%), and C58 (54.60 ± 1.27 and 55.90 ± 1.56%) showed relatively high hydrophobicity to toluene and xylene, respectively. These strains also displayed high autoaggregation of C3 (69.30 ± 1.10%), C18 (73.40 ± 1.33%), C20 (64.20 ± 1.50%), C39 (55.90 ± 1.62%), and C58 (53.70 ± 1.10%) following a 24-h incubation. According to Yasmin et al. (2020), who studied the cell surface properties, *Bifidobacterium longum* B-11 (78.89%) and *B. longum* B-5 (74.21%) had the highest hydrophobicity values. In addition, Kouhi et al. also examined 10 LAB strains for their ability to autoaggregate and cell surface hydrophobicity^21^.

#### Cholesterol absorption

Table 3 shows the results of the cholesterol-lowering test. The results obtained showed that the C3 and C18 strains had the highest cholesterol absorption rates at 67.20 ± 1.21 and 65.10 ± 1.10%, and it was also shown that the C58 strain had the lowest cholesterol absorption rate of 09.20 ± 0.64% among the strains studied. High cholesterol has several unfavorable health consequences, including an increased risk of cardiovascular disease, the leading cause of death. Hypocholesterolemia is a major risk factor for the development of coronary heart disease. Blood cholesterol levels must be decreased to prevent the condition. When used to treat hypercholesterolemia, the medication causes a variety of negative effects^20^. Therefore, it is crucial to reduce serum cholesterol using natural, affordable methods. In order to reduce their reliance on pharmacological therapy, consumers are more interested in utilizing safe alternative products for hypercholesterolemia. In this respect, one of the most effective and promising strategies for lowering blood cholesterol is the use of probiotic-containing dietary supplements. Cholesterol absorption into the cell membrane, cholesterol uptake during growth, and cholesterol adhesion to the cell surface are some of the hypothesized methods for how probiotic bacteria lower cholesterol^28^. A similar cholesterol reduction trend was reported previously when strains were isolated from Motal cheese^6^ and Tarkhineh products^12^. According to the current study's findings, different *Lactobacillus* strains can lower cholesterol levels.

#### Antimicrobial assay

The antimicrobial activity of the isolates evaluated against a range of harmful bacteria is shown in Table 4. According to the findings, all five examined microbes demonstrated a broad spectrum of antagonistic activity against the pathogens studied. In the present study, the inhibitory effect of different concentrations of silymarin extract in DMSO + PEG solvent and also in methanol solvent on the target pathogens was examined, and the outcomes obtained are shown in Table 5. The findings obtained showed that silymarin extract inhibited the growth of the pathogens studied in a dose-dependent manner. The results showed that silymarin extract in DMSO + PEG solvent has stronger inhibitory potency than methanol solvent. Silymarin extract in DMSO + PEG solvent showed an inhibitory effect on all pathogens tested at all concentrations, but in methanol solvent it had an inhibitory effect only up to a concentration of 3000 mg mL^−1^ and only on some pathogens. In addition, silymarin extract in methanol had no impact on *S. sanguinis, L. monocytogenes, S. flexneri,* or* E. coli.*

**Table 4:** The inhibitory effect of isolated LAB strains against pathogens.

Pathogens	Origin	Incubation conditions	Diameter of inhibition zone (mm)					
			C3	C18	C20	C39	C58	CON
*S. sanguinis*	PTCC 1449	37 °C in TSA II medium	13.6 ± 0.3^c^	19.0 ± 0.2^a^	16.1 ± 0.2^b^	12.3 ± 0.6^d^	18.9 ± 0.5^a^	0.0 ± 0.0^e^
*S. salivarius*	PTCC 1448	37 °C in TSA II medium	16.3 ± 0.4^c^	20.9 ± 0.5^a^	16.7 ± 0.2^c^	18.8 ± 0.5^b^	21.3 ± 0.8^a^	0.0 ± 0.0^d^
*S. sobrinus*	PTCC 1601	37 °C in Blood agar medium	18.3 ± 0.7^b^	21.6 ± 0.4^a^	17.2 ± 0.5^c^	17.4 ± 0.4^bc^	21.2 ± 0.9^a^	0.0 ± 0.0^d^
*Y. enterocolitica*	ATCC 23,715	37 °C in TSA medium	15.8 ± 0.4^d^	20.9 ± 0.4^a^	16.4 ± 1.1^d^	17.8 ± 0.8^c^	19.3 ± 0.9^b^	0.0 ± 0.0^e^
*S. mutans*	PTCC 1683	37 °C in MHA medium	15.7 ± 0.4^b^	25.4 ± 0.6^a^	14.2 ± 0.4^c^	13.3 ± 0.2^d^	14.4 ± 0.3^c^	0.0 ± 0.0^e^
*P.aeruginosa*	PTCC 1181	26 °C in Nutrient agar medium	14.3 ± 0.4^c^	21.5 ± 0.4^a^	15.7 ± 0.2^b^	14.3 ± 0.3^c^	14.6 ± 0.4^c^	0.0 ± 0.0^d^
*S. aureus*	ATCC 25,923	37 °C in Blood agar medium	15.7 ± 0.3^c^	21.8 ± 0.4^a^	16.4 ± 0.6^b^	15.3 ± 0.4^c^	21.7 ± 0.2^a^	0.0 ± 0.0^d^
*B. subtilis*	ATCC 19,652	30 °C in Nutrient agar/broth medium	15.1 ± 0.3^c^	21.6 ± 0.3^a^	12.3 ± 0.6^d^	16.3 ± 0.4^b^	21.5 ± 0.1^a^	0.0 ± 0.0^e^
*L. monocytogenes*	ATCC 13,932	37 °C in BHI medium	16.2 ± 0.4^d^	18.8 ± 0.6^c^	15.7 ± 0.2^d^	20.0 ± 0.5^b^	20.9 ± 0.5^a^	0.0 ± 0.0^e^
*K. pneumoniae*	PTCC 1053	37 °C in MPA medium	20.2 ± 0.4^b^	18.7 ± 0.2^d^	16.1 ± 0.2^e^	19.7 ± 0.3^c^	21.0 ± 0.4^a^	0.0 ± 0.0^f^
*S. flexneri*	PTCC 1234	37 °C in MHA medium	18.6 ± 0.3^b^	23.8 ± 0.7^a^	14.1 ± 0.2^d^	15.2 ± 0.4^c^	19.0 ± 0.4^b^	0.0 ± 0.0^e^
*E. coli*	PTCC 1276	37 °C in LB medium	15.8 ± 0.5^c^	20.9 ± 0.4^a^	16.8 ± 0.5^b^	10.2 ± 0.4^d^	20.8 ± 0.3^a^	0.0 ± 0.0^e^

Values are mean ± standard error of triplicates. ^a-f^ Means in the same row with different lowercase letters differed significantly (*P* < 0.05).

ATCC: American Type Culture Collection, Virginia, USA. PTCC: Persian Type Culture Collection, Tehran, Iran.

*Streptococcus sanguinis, Streptococcus salivarius, Streptococcus sobrinus, Yersinia enterocolitica, Streptococcus mutans, Pseudomonas aeruginosa, Staphylococcus aureus, Bacillus subtilis, Listeria monocytogenes, Klebsiella pneumoniae, Shigella flexneri, and Escherichia coli*. These pathogenic organisms were purchased from the Persian Type Culture Collection (PTCC).

**Table 5:** Inhibitory effect of different concentrations of silymarin extract in DMSO + PEG and methanol solvent against pathogens.

Pathogens	375	750	1500	3000	6000	9000	CON
Diameter of inhibition zone (mm) of silymarin extract in DMSO + PEG solvent							
*S. sanguinis*	8.5 ± 0.2^f^	10.1 ± 0.1^e^	11.3 ± 0.3^d^	12.5 ± 0.2^c^	14.3 ± 0.2^b^	17.8 ± 0.1^a^	0.0 ± 0.0^g^
*S. salivarius*	7.5 ± 0.2^f^	8.3 ± 0.2^e^	9.5 ± 0.2^d^	12.3 ± 0.1^c^	14.5 ± 0.3^b^	16.5 ± 0.1^a^	0.0 ± 0.0^g^
*S. sobrinus*	9.4 ± 0.2^f^	10.4 ± 0.4^e^	13.4 ± 0.3^d^	15.5 ± 0.3^c^	17.5 ± 0.2^b^	18.7 ± 0.3^a^	0.0 ± 0.0^g^
*Y. enterocolitica*	10.3 ± 0.2^f^	11.4 ± 0.2^e^	12.4 ± 0.3^d^	13.3 ± 0.3^c^	14.3 ± 0.1^b^	15.2 ± 0.1^a^	10.1 ± 0.2^f^
*S. mutans*	8.5 ± 0.1^f^	9.3 ± 0.1^e^	10.5 ± 0.2^d^	11.5 ± 0.2^c^	12.5 ± 0.3^b^	14.6 ± 0.3^a^	0.0 ± 0.0^g^
*P.aeruginosa*	5.4 ± 0.3^f^	6.6 ± 0.3^e^	7.4 ± 0.3^d^	10.4 ± 0.4^c^	12.3 ± 0.1^b^	14.5 ± 0.2^a^	0.0 ± 0.0^g^
*S. aureus*	8.3 ± 0.2^f^	10.5 ± 0.1^e^	11.4 ± 0.3^d^	12.2 ± 0.1^c^	13.4 ± 0.3^b^	15.6 ± 0.2^a^	0.0 ± 0.0^g^
*B. subtilis*	6.5 ± 0.1^f^	8.6 ± 0.2^e^	10.4 ± 0.1^d^	13.5 ± 0.2^c^	15.2 ± 0.1^b^	18.6 ± 0.3^a^	0.0 ± 0.0^g^
*L. monocytogenes*	5.5 ± 0.1^f^	6.3 ± 0.3^e^	8.3 ± 0.2^d^	10.4 ± 0.1^c^	12.4 ± 0.3^b^	14.7 ± 0.1^a^	0.0 ± 0.0^g^
*K. pneumoniae*	7.6 ± 0.2^f^	8.5 ± 0.3^e^	10.4 ± 0.1^d^	12.5 ± 0.4^c^	13.5 ± 0.2^b^	15.4 ± 0.3^a^	0.0 ± 0.0^g^
*S. flexneri*	7.6 ± 0.1^f^	8.5 ± 0.3^e^	10.2 ± 0.2^d^	12.5 ± 0.2^c^	14.3 ± 0.1^b^	17.7 ± 0.1^a^	0.0 ± 0.0^g^
*E. coli*	8.6 ± 0.2^f^	10.6 ± 0.3^e^	12.2 ± 0.1^d^	15.6 ± 0.1^c^	17.5 ± 0.2^b^	19.5 ± 0.2^a^	0.0 ± 0.0^g^
Diameter of inhibition zone (mm) of silymarin extract in methanol solvent							
*S. sanguinis*	0.0 ± 0.0^a^	0.0 ± 0.0^a^	0.0 ± 0.0^a^	0.0 ± 0.0^a^	0.0 ± 0.0^a^	0.0 ± 0.0^a^	0.0 ± 0.0^a^
*S. salivarius*	0.0 ± 0.0^d^	6.1 ± 0.1^c^	8.5 ± 0.2^b^	9.4 ± 0.3^a^	0.0 ± 0.0^d^	0.0 ± 0.0^d^	0.0 ± 0.0^d^
*S. sobrinus*	0.0 ± 0.0^a^	0.0 ± 0.0^a^	0.0 ± 0.0^a^	0.0 ± 0.0^a^	0.0 ± 0.0^a^	0.0 ± 0.0^a^	0.0 ± 0.0^a^
*Y. enterocolitica*	8.4 ± 0.2^d^	10.7 ± 0.2^c^	11.6 ± 0.3^b^	12.1 ± 0.1^a^	0.0 ± 0.0^e^	0.0 ± 0.0^e^	0.0 ± 0.0^e^
*S. mutans*	6.5 ± 0.2^d^	7.7 ± 0.2^c^	8.2 ± 0.3^b^	10.8 ± 0.1^a^	0.0 ± 0.0^e^	0.0 ± 0.0^e^	0.0 ± 0.0^e^
*P.aeruginosa*	6.2 ± 0.2^d^	7.6 ± 0.3^c^	8.4 ± 0.4^b^	10.6 ± 0.1^a^	0.0 ± 0.0^e^	0.0 ± 0.0^e^	0.0 ± 0.0^e^
*S. aureus*	7.2 ± 0.2^d^	9.7 ± 0.2^c^	10.4 ± 0.2^b^	11.5 ± 0.3^a^	0.0 ± 0.0^e^	0.0 ± 0.0^e^	0.0 ± 0.0^e^
*B. subtilis*	5.2 ± 0.3^d^	6.7 ± 0.1^c^	7.3 ± 0.4^b^	8.3 ± 0.4^a^	0.0 ± 0.0^e^	0.0 ± 0.0^e^	0.0 ± 0.0^e^
*L. monocytogenes*	0.0 ± 0.0^a^	0.0 ± 0.0^a^	0.0 ± 0.0^a^	0.0 ± 0.0^a^	0.0 ± 0.0^a^	0.0 ± 0.0^a^	0.0 ± 0.0^a^
*K. pneumoniae*	7.2 ± 0.2^d^	10.6 ± 0.4^c^	11.3 ± 0.3^b^	12.5 ± 0.3^a^	0.0 ± 0.0^e^	0.0 ± 0.0^e^	0.0 ± 0.0^e^
*S. flexneri*	0.0 ± 0.0^a^	0.0 ± 0.0^a^	0.0 ± 0.0^a^	0.0 ± 0.0^a^	0.0 ± 0.0^a^	0.0 ± 0.0^a^	0.0 ± 0.0^a^
*E. coli*	0.0 ± 0.0^a^	0.0 ± 0.0^a^	0.0 ± 0.0^a^	0.0 ± 0.0^a^	0.0 ± 0.0^a^	0.0 ± 0.0^a^	0.0 ± 0.0^a^

Notes: Values are mean ± standard error of triplicates. ^a-g^ Means in the same row with different lowercase letters differed significantly (P < 0.05). CON: DMSO + PEG and methanol solvent.

GIT infections can be treated with probiotics^29^. Antimicrobial peptides may be one of the most efficient defenses against contagious diseases. Bacteriocin, organic acids, and hydrogen peroxide produced by LAB have been hypothesized to combat pathogenic microorganisms^30,31^. The outcome showed that the five strains that were put to the test would be good candidates for use in food preservation. Probiotic Lactobacillus has been shown in a similar investigation by Acharjee et al. to have antibacterial action against a variety of pathogenic pathogens^32^. Similarly, our study demonstrated different inhibition zone diameters by extracting *Lactobacillus* species. Consistent with this study, Haghshenas et al. stated that lactobacilli isolated from Iranian fermented milk products exhibited good and potent antibacterial activity against some tested pathogenic microbes^33^.

#### Antifungal activity

**Figure 1:**
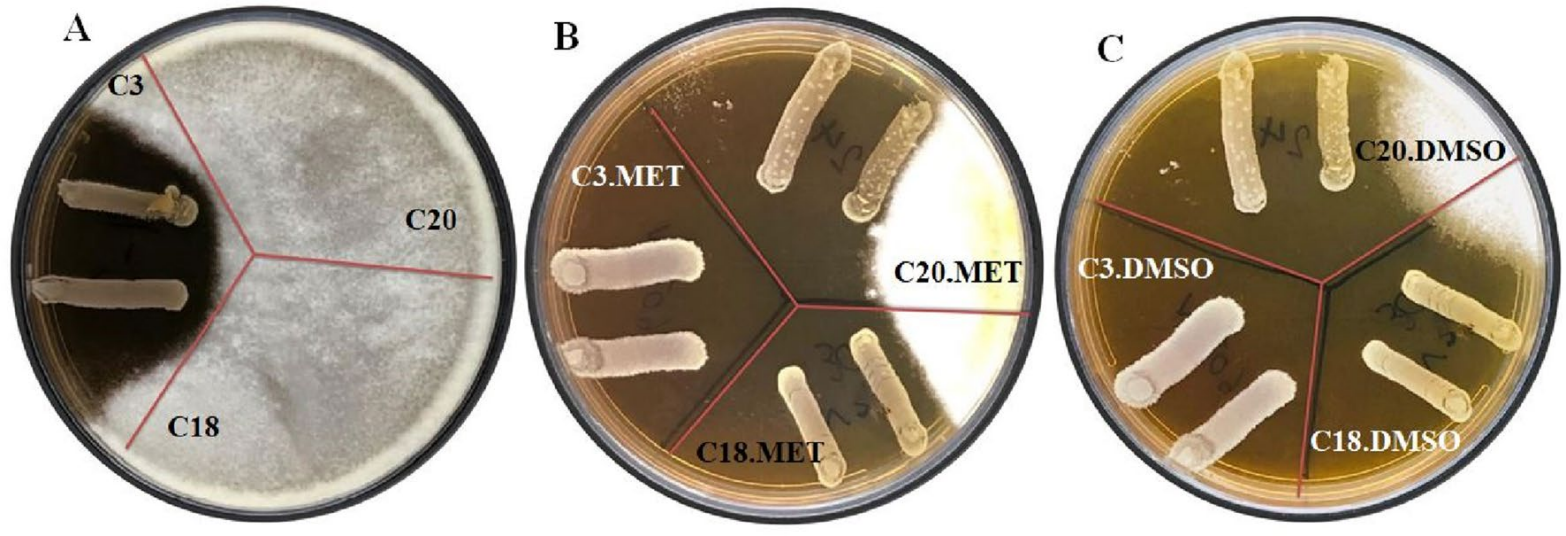
Antifungal activity of potential probiotic strains C3, C18 and C20 on *A. flavus* (Panel **A**); potential probiotic strains along with silymarin extract in methanol (Panel **B**); potential probiotic strains along with silymarin extract in DMSO + MEG (Panel **C**).

To investigate the antifungal properties of silymarin extract and potentially probiotic bacterial strains, *A. flavus* was used in this work. For this purpose, the antifungal effects of the potentially probiotic strains obtained were studied alone and in combination with silymarin extract in DMSO + PEG and methanol solvents. The outcomes of the antifungal activity of strains are presented in Fig. 1. Based on the results, strains C18, C20, C39, and C58 had no inhibitory effects on *A. flavus* (results not shown), while strain C3 showed remarkable inhibitory effects (Fig. 1, panels A). The results of the simultaneous antifungal activity of potentially probiotic strains and silymarin showed that the combination of silymarin extract and probiotic strains had a greater antifungal effect than the control group as well as the probiotic group alone. Interestingly, in addition to strain C3, strains C18, and C20 also showed inhibitory effects against *A. flavus* when strains with silymarin extract in methanol were used at the same time (Fig. 1, Panels B). In addition, silymarin extract in DMSO + PEG increased the antagonistic activity of strains C3, C18 and C20 (Fig. 1, Panels C).

Bacterial infections contribute to the mortality toll worldwide, particularly in developing nations where poverty is rife. Human fungal infections, which operate as hidden killers, make the situation worse^34^. In contrast, owing to the advent of multidrug-resistant pathogens and the waning efficacy of current medicines, the morbidity and mortality rates of the global population are rising at a concerning rate^32^. Utilizing antimicrobial and antifungal compounds derived from natural sources to improve the efficacy and potency of currently available antibiotics while also eliminating their negative side effects is urgently required at this time^35^. The antifungal and antibacterial activities of a few *Lactobacillus* strains isolated from milk curd products, as well as their synergistic effects when combined with silymarin, were investigated in this work. The probiotics demonstrated antifungal and antimicrobial activity against the pathogens tested in this study. The efficacy of silymarin was also observed, implying that silymarin could enhance probiotic antifungal activity while also acting as a prebiotic. The findings of this study corroborate those of Abouloifa et al., who discovered that lactobacilli isolated from fermented natural green olives had antifungal activity against a diverse range of molds (*Penicillium digitatum, Fusarium oxysporum, Aspergillus niger,* and *Rhizopus sp*.) and yeasts (*Rhodotorula *sp. and *Candida pelliculosa*)^36^.

### Safety aspects

#### Hemolysis activity

Table 3 displays the hemolytic activity outcomes in the isolated strains. According to the results, all of the strains obtained have γ-hemolytic activity, and no complete hemolysis was observed in the isolated strains. The European Food Safety Authority (EFSA) recommends assessing hemolytic activity when isolated bacteria are intended for use in food, even if they have GRAS status^37^. In the current study, the hemolytic potential of five lactobacilli was tested on blood agar plates. None of the isolates tested showed β-hemolytic or α-hemolytic activity when grown on blood agar. Our findings were comparable to those of other studies^6,18,21^.

#### Antibiotic susceptibility

Table 6 shows the antibiotic susceptibility of strains. The antibiotic susceptibility of the selected strains was tested using cefixime (15 mg), azithromycin (30 mg), amoxicillin (20 mg), doxycycline (15 mg), trimethoprim-sulfamethoxazole (30 mg), cephalexin (30 mg), ciprofloxacin (20 mg), amoxicillin clavulanic acid (30 mg), and vancomycin (30 mg). All isolates were susceptible to the majority of tested antibiotics, with the exception of vancomycin, to which they were resistant. Four of the five isolates were resistant to ciprofloxacin, but strain C18 was susceptible. *Lactobacillus* bacteria have been shown in the literature to be antibiotic-resistant and can be safely consumed after antibiotic therapy to help maintain gut flora balance^17,38^. Resistance to vancomycin was comparable to previously described LAB strains^28,39^. *Lactobacillus* antibiotic resistance is intrinsic and not transmissible, so the corresponding genes are not transferred to pathogenic organisms^39^.

**Table 6:** Antibiotic susceptibility profiles of isolated LAB strains.

Isolated strains	Antibiotics								
	CFM	AZM	AMX	D	SXT	CP	CN	AMC	V
C3	20 (S)	16 (I)	28 (S)	38 (S)	32 (S)	0 (R)	32 (S)	41 (S)	0 (R)
C18	0 (R)	26 (S)	25 (S)	38 (S)	50 (S)	15 (R)	0 (R)	32 (S)	0 (R)
C20	21 (S)	21 (S)	38 (S)	35 (S)	42 (S)	0 (R)	35 (S)	46 (S)	0 (R)
C39	26 (S)	23 (S)	45 (S)	35 (S)	45 (S)	0 (R)	41 (S)	40 (S)	0 (R)
C58	0 (R)	0 (R)	0 (R)	0 (R)	0 (R)	0 (R)	0 (R)	0 (R)	0 (R)

CFM, cefixime; AZM, azithromycin; AMX, amoxicillin; D, doxycycline; SXT, trimethoprim sulfamethoxazole; CP, ciprofloxacin; CN, cephalexin; AMC, amoxicillin-clavulanic acid; V, vancomycin.

Cefixime results based on R ≤ 15 mm; I: 16–18 mm; S ≥ 19 mm.

Azithromycin results based on R ≤ 13 mm; I: 14–17 mm; S ≥ 18 mm.

Amoxicillin results based on R ≤ 18 mm; I: 19–21 mm; S ≥ 22 mm.

Doxycycline results based on R ≤ 10 mm; I: 11–13 mm; S ≥ 14 mm.

Trimethoprim sulfamethoxazole results based on R ≤ 25 mm; I: 26–29 mm; S ≥ 30 mm.

Ciprofloxacin results based on R ≤ 15 mm; I: 16–20 mm; S ≥ 21 mm.

Cephalexin results based on R ≤ 14 mm; I: 15–17 mm; S ≥ 18 mm.

Amoxicillin-clavulanic acid results based on R ≤ 13 mm; I: 14–17 mm; S ≥ 18 mm.

Vancomycin results based on R ≤ 14 mm; I: 15–16 mm; S ≥ 17 mm.

(Performance Standards for Antimicrobial Susceptibility Testing, from Clinical and Laboratory Standards Institute, Twenty-Third Informational Supplement, Wayne, PA (CLSI 2013).

### Molecular identification and DNA sequencing

The morphological profile of the chosen LAB isolates was confirmed using 16S rRNA gene sequencing. The amplification of the 16S rRNA genes showed that all five isolates were *Lactobacillus*. Strains C3 and C18 belonged to *Limosilactobacillus fermentum*, strains C20 belonged to *Lactiplantibacillus pentosus,* and strains C39 and C58 belonged to *Lactiplantibacillus plantarum*. These five strains were entered into the NCBI GeneBank with accession numbers OP317516, OP317547, OP324655, OP317548, and OP317549, respectively.

## Conclusion

In conclusion, the 52 LAB strains from traditional Iranian curd were identified as different *Lactobacillus*. Five of the 52 LAB strains showed high acid and bile salt resistance, hydrophobicity, autoaggregation, coaggregation, cholesterol-removing ability, and significant antifungal and antimicrobial activity against *A. flavus* and some pathogenic bacteria. They were also highly susceptible to amoxicillin, doxycycline, and amoxicillin-clavulanic acid. These findings suggest that the isolated *Lactobacillus* strains, including *L. fermentum* C3, *L. fermentum* C18, *L. pentosus* C20, *L. plantarum* C39, and *L. plantarum* C58, in combination with silymarin extract, are excellent candidates for food use. The findings of the current study showed that probiotic *Lactobacillus* strains isolated from traditional dairy products such as curd and silymarin have the potential to curb the *A. flavus* infection. The results can indicate that foods enriched with specific indigenous probiotic microorganisms and plant extracts (*M. silybum*) would reduce the toxicity risk of aflatoxins in foodstuffs. More research is needed, however, to investigate the probiotic features of various *Lactobacillus* strains.

## Data availability

The datasets generated and/or analysed during the current study are available in the NCBI GeneBank repository, accession numbers OP317516, OP317547, OP324655, OP317548 and OP317549.
